# Overview and Evolution of Insect Fibroin Heavy Chain (FibH)

**DOI:** 10.3390/ijms25137179

**Published:** 2024-06-29

**Authors:** Tong Zhang, Sanyuan Ma, Ziyang Zhang, Yongkang Guo, Daiying Yang, Wei Lu

**Affiliations:** 1 Integrative Science Center of Germplasm Creation in Western China (CHONGQING) Science City, Biological Science Research Center, Southwest University, Chongqing 400715, China; zt137703197@email.swu.edu.cn (T.Z.); masy@swu.edu.cn (S.M.); gyk666@email.swu.edu.cn (Y.G.); dm123654@email.swu.edu.cn (D.Y.); 2 College of Sericulture, Textile and Biomass Sciences, Southwest University, Chongqing 400715, China; ziyangzhang0416@163.com

**Keywords:** Lepidoptera, Trichoptera, fibroin heavy chain, evolution

## Abstract

The *FibH* gene, crucial for silk spinning in insects, encodes a protein that significantly influences silk fiber mechanics. Due to its large size and repetitive sequences, limited known sequences of insect FibH impede comprehensive understanding. Here, we analyzed 114 complete *FibH* gene sequences from Lepidoptera (71 moths, 24 butterflies) and 13 Trichoptera, revealing single-copy *FibH* in most species, with 2–3 copies in Hesperinae and Heteropterinae (subfamily of skippers). All *FibH* genes are structured with two exons and one intron (39–45 bp), with the second exon being notably longer. Moths exhibit higher GC content in *FibH* compared to butterflies and Trichoptera. The FibH composition varies among species, with moths and butterflies favoring Ala, Gly, Ser, Pro, Gln, and Asn, while Trichoptera FibH is enriched in Gly, Ser, and Arg, and has less Ala. Unique to Trichoptera FibH are Tyr, Val, Arg, and Trp, whereas Lepidoptera FibH is marked by polyAla (polyalanine), polySer (polyserine), and the hexapeptide GAGSGA. A phylogenetic analysis suggests that Lepidoptera FibH evolved from Trichoptera, with skipper FibH evolving from Papilionoidea. This study substantially expands the FibH repertoire, providing a foundation for the development of artificial silk.

## 1. Introduction

Unrivaled in their adaptability and ubiquity, insects serve as a cornerstone of Earth’s biodiversity and play a crucial role in ecosystems as decomposers, pollinators, and food. Beyond their ecological significance, certain insects synthesize silk proteins to construct various marvelous structures, including a cocoon, nest, and egg case [1]. Silkworm (*Bombyx mori*) has the unique ability to spin silk, a sustainable biomaterial captivating human attention for millennia due to its distinctive physical and chemical properties [2].

Insects are renowned for their silk production, with moths and butterflies being the most common silk-producing insects. Other fascinating orders such as Trichoptera and Hymenoptera including bees, wasps, and ants are also known for their silk-spinning abilities [1,3,4]. Silk is an impressive and versatile biopolymer, primarily composed of proteins interwoven with metal ions, and other inorganic and organic substances. These components vary across species, resulting in silk with distinct characteristics. Silkworm cocoon silk is predominantly made up of sericin and fibroin including FibH, FibL, and P25/fibrohexamerin [5,6]. The oriental moth cocoon is composed of the most abundant asparagine-rich proteins, fibroin, mineral crystals, and organic substances [7]. The caddisfly silk contains FibH, FibL, endoplasmin, serpin, chymotrypsin-like protease, two mucin-like proteins, pseudofibrin, and three additional unannotated proteins without P25 [8].

The FibH is a fundamental component of insect silk, nearly universally present in the silk of Lepidoptera and Trichoptera. The FibH of *Bombyx mori* (*BmorFibH*) is encoded by the fibroin heavy chain gene nearly 17 kb in length, with a molecular weight of 350 kDa [5]. Based on sequence characteristics, BmorFibH is divided into N- and C-terminal non-repetitive domains (NTDs and CTDs, respectively), enclosing a repetitive core (RC). The NTD and CTD are involved in the folding, assembly, and secretion of silk proteins, and extremely conserved across silkworm strains [9,10,11,12,13,14]. The RC is composed of many tandem repetitive sequences [13,15,16]. Under the synergistic effect of many factors such as pH, metal ions, and shear force, the RC forms the crystalline region, which confers physical properties to the silk fiber [17,18,19,20]. Despite having the same repetitive motifs, such as hexapeptide GAGAGS and GAGAGY, the number and distribution of these motifs vary, leading to significant differences in the FibH sequences across silkworm strains [13,16].

The FibH is a pivotal determinant in the mechanical properties of insect silk fibers [21,22]. Deciphering and expanding the repertoire of the *FibH* gene promise to shed light on the intricate molecular evolution of insect silk [23]. This knowledge is critical for the synthetic replication of silk’s unique properties [24]. Furthermore, understanding the diversity of FibH can shed light on the pathways by which nature fine-tunes these proteins for a specific function. This revelation could spark innovative methodologies in the design of silk-based materials, enabling tailoring the properties of silk fiber to a specific application, such as achieving superior mechanical properties and flexibility. To date, the complete *FibH* sequences of about 280 mulberry silkworms (*Bombyx mori* and *Bombyx mandarina*) and six other insect species have been deciphered, revealing a vast and unexplored landscape of *FibH* diversity [13,16]. However, the complete *FibH* sequences of countless insects remain unknown, presenting a formidable challenge to our quest for a holistic grasp of silk’s evolutionary history and its functional diversity across the insect kingdom.

In this study, we undertook a comprehensive analysis of publicly available Lepidoptera and Trichoptera genomic data, focusing on extracting the complete *FibH* sequence through a homology analysis. We systematically examined the characteristics of all the FibH sequences, including unique amino acid composition, repetitive motifs, and evolutionary relationships. Our work can substantially enrich our grasp of the insect silk diversity. Moreover, it will establish a solid theoretical framework for the innovation and synthesis of novel artificial silk, thereby opening new avenues in the field of biomaterial science.

## 2. Results

### 2.1. FibH Gene Copy Number Variation Occurs Only in Skipper (Hesperiidae)

**Figure 1:**
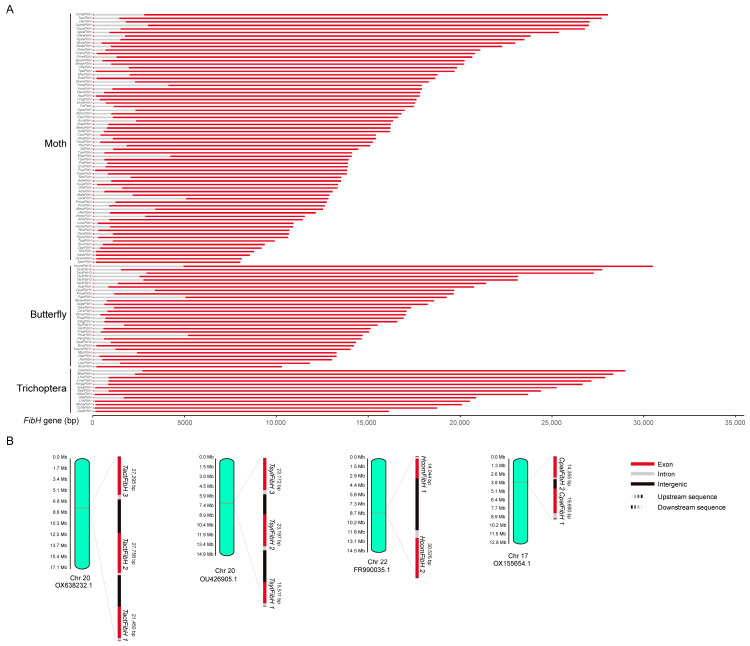
The overview and copy number variation of the insect *FibH* gene. (**A**) The *FibH* genes of Trichoptera, moths, and butterflies. (**B**) The copy number variation of the *FibH* gene in skippers.

Using publicly available genomic data, we extracted 101 complete *FibH* gene sequences from Lepidoptera, involving 40 families. Limited by the available assembled genomic data, we only extracted 13 complete *FibH* gene sequences from four families of Trichoptera. The *FibH* gene of Lepidoptera is 8040 (*AperFibH*)-30,526 bp (*HcomFibH 2*) long. The *FibH* gene of Trichoptera varied between 16,133 bp (*OalbFibH*) and 28,991 bp (*CdisFibH*). All insect *FibH* genes consist of two exons separated by a single intron. Exon 1 is relatively short, ranging from 39 to 45 bp, while exon 2 is between 7 and 29 kb and varies greatly among different species. The introns of all *FibH* genes range from 73 to 5159 bp, and their guanine–cytosine (GC) content is around 50% (Figure 1A and Figure S1).

The *FibH* in Trichoptera and moths is a single-copy gene. Interestingly, there is copy number variation (CNV) in the *FibH* gene of skippers. Both *Thymelicus acteon* and *Thymelicus sylvestris* have three copies of the *FibH* gene, and they are located on chromosome 20. The lengths of the TactFibH (*TactFibH 1*, *2*, and *3*) and TsylFibH (*TsylFibH 1*, *2*, and *3*) genes are 21,450 bp, 27,758 bp, 27,295 bp, 15,511 bp, 23,197 bp, and 23,172 bp, respectively. *Hesperia comma* and *Carterocephalus palaemon* each have two *FibH* genes located on chromosomes 22 and 17, respectively. The *HcomFibH 1* and *2*, as well as *CpalFibH 1* and *2*, have 14,044, 30,526, 19,690, and 14,355 base pairs, respectively (Figure 1B, Table 1).

### 2.2. FibH Differs in Length, Amino Acid Composition, and Properties among Insects

**Figure 2:**
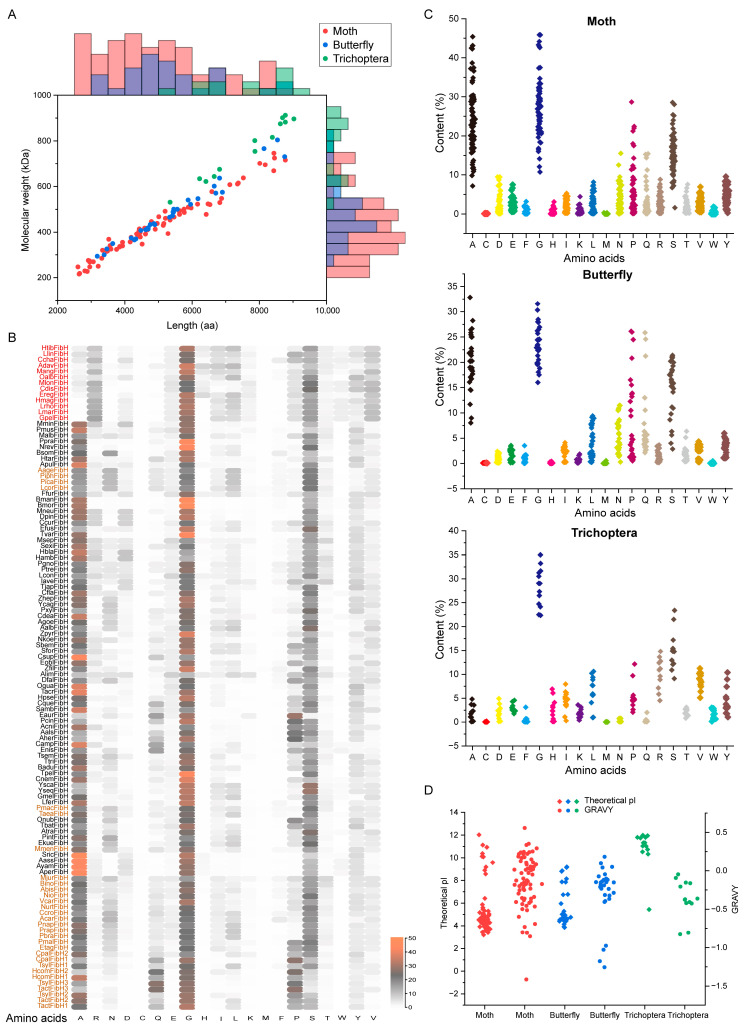
The length, molecular weight, composition, and properties of FibH. (**A**) The length and molecular weight of FibH. (**B**) The amino acid composition of each insect FibH. The red, black, and orange colors represent the FibH of Trichoptera, moth, and butterfly, respectively. (**C**) The amino acid composition of moth, butterfly, and Trichoptera FibH. (**D**) The theoretical isoelectric point (pI) and grand average of hydropathicity (GRAVY) of FibH.

The protein sequences of all *FibH* genes have been deduced. The length and molecular weight of FibH in moths vary significantly, ranging from 2603 (IaveFibH) to 8773 (TacrFibH) amino acids (with an average of 5006), with 216.05948–746.16907 kDa. Butterflies have FibH of equal length to moths, between 3178 and 8754 (average: 5420). Skippers with multiple copies have their FibH distributed between 4284 (HcomFibH 1) and 8754 (TactFibH 2) (Table 1). In contrast, the FibH of Trichoptera is generally longer and has a larger molecular weight than that of the Lepidoptera, with 5344–9027 amino acids (average: 7650), and 531.07887–912.29248 kDa. Ala, Gly, Ser, Pro, Gln, and Asn are the most abundant in Lepidoptera FibH, while Trichoptera FibH is rich in Gly, Ser, and Arg, with only less Ala (Figure 2A–C, Table 1). Based on the complexity of the sequence and domain, the FibH is divided into the conserved NTD, CTD, and highly variable repetitive core (RC). The NTD of BmorFibH contains 151 amino acids, with the first 14 amino acids encoded by exon 1, and the first 18 amino acids being a signal peptide, which is the same as that in skippers (Table 1). Gly 1, Ile 8, and Gly 52 are three conserved sites that are present in all NTDs. In the NTD of Trichoptera FibH, the sixth and seventh amino acids are LL (LeuLeu). The CTD of BmorFibH contains 58 amino acids, and the 20th residue (Cys-c20) from the C-terminus forms a disulfide bond with FibL, which is also conserved and present in the CTD of non-Saturniidae FibH (Figure S2).

To investigate the effects of amino acid composition on the properties of FibH, the isoelectric points (pI) and grand average of hydropathicity (GRAVY) of all FibHs were analyzed. The pI of moth FibH is distributed between 3.2 and 12.3, with 85.9155% being less than 7; the GRAVY scores range from −1.415 to 0.557, with hydrophilic FibH constituting 57.7465%. For butterfly FibH, the pI is distributed between 3.8 and 9.19, with 76.6667% of the pI values being less than 7; the GRAVY scores are between −1.256 and 0.183, with hydrophilic FibH making up 86.6667%. Compared to moths and butterflies, the pI and GRAVY of Trichoptera (caddisfly) FibH show more pronounced differences. Except for OalbFibH, which has a pI less than 7, the pI of other Trichoptera FibH ranges from 10.33 to 11.99. All Trichoptera FibH are hydrophilic proteins with an average hydrophobicity ranging from −0.827 to −0.048, which is likely closely related to their living environments (Figure 2D). In skippers, different copies of FibH exhibit varying properties. The pI values for both HcomFibH 1 and HcomFibH 2 are above 8, with GRAVY scores of −0.288 and −1.029, respectively. Conversely, the pI of CpalFibH 1 and CpalFibH 2 are both less than 7, and their GRAVY scores are −0.977 and −0.395, respectively. Interestingly, the pI values for TactFibH 1 and TsyFibH 1 are 4.89 and 5.29, respectively, with GRAVY scores of −0.323 and −0.104. The pI values for the other copies are all around 8, and both TactFibH 3 and TsyFibH 3 are highly hydrophilic (Table 1). The different chemical properties of the various FibH copies suggest that the mechanical properties of this skipper silk may also differ.

### 2.3. Insect FibH Has Developed Unique Motifs during Evolution

**Figure 3:**
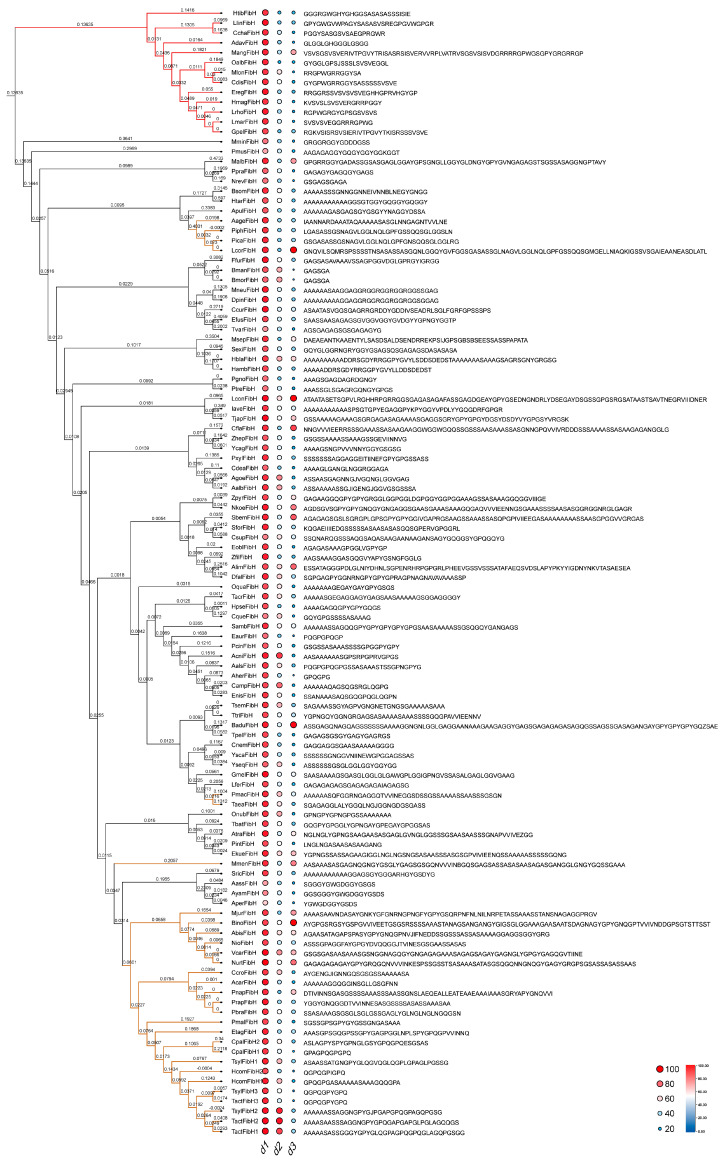
The phylogenetic tree of insect FibH and their motifs. d1 represents the proportion of all motifs to the total sequence length, with a median of 0.89; d2 indicates the proportion of the most prevalent motif (the typical motif) to the total sequence length, with a median of 0.50; d3 denotes the number of amino acid residues in the typical motif, with a median of 35.64. The amino acid sequence on the right represents the motif sequence with the highest percentage in each insect FibH. The red, black, and orange colors in the phylogenetic tree represent the FibH of Trichoptera, moth, and butterfly, respectively.

The fibroin heavy chain is divided into the NTD, CTD, and repetitive region (RC), which constitutes approximately 85% of FibH and is composed of numerous motifs. FibH is the core protein of insect silk, and its evolutionary direction is consistent with that of insect silk fibers. To elucidate the evolutionary history of FibH, the phylogenetic relationships of all FibH were analyzed. In the phylogenetic tree, the Trichoptera (caddisfly) FibH forms a separate branch and possesses typical motifs distinct from those of the Lepidoptera (moth and butterfly) (Figure 3 and Figure S3). The amino acids Tyr, Val, Arg, and Trp are unique to the majority of Trichoptera FibH. The phylogenetic tree indicates that MminFibH and PmusFibH are ancestral to other Lepidoptera FibH, from which many branches have evolved. There are differences in the typical motifs among different Lepidoptera insects. PolyAla, polySer, and the hexapeptide GAGSGA are ubiquitously present in Lepidoptera FibH and represent peptides that distinguish Lepidoptera FibH motifs from those of Trichoptera FibH. It is well known that GAGSGA is a typical motif of the BmorFibH [13,15]. In fact, apart from the silkworm, other species in the Bombycidae family, such as TvarFibH, and in the Notodontidae and Psychidae family, such as FfurFibH and LferFibH, also contain numerous GAGSGA motifs (Figure 3, Table S1).

Butterfly FibH is believed to have evolved from moth FibH, which is consistent with the conclusion that moths are the ancestors of butterflies. The FibH of skippers (a subfamily of butterflies) evolved from that of Papilionoidea, and multiple copies of FibH have evolved from single-copy FibH, such as PmalFibH and EtagFibH (Figure S3). Interestingly, there are significant differences in the motif of different FibH copies. While they possess different motifs, all contain the tripeptide QGP. TactFibH 1 and 2 and TsylFibH 2 have similar motifs that include polyAla and GPQ. TactFibH 3 and TsylFibH 3 share the same typical motif, QGPQGPYGPQ. Other FibH have different motifs, and all contain the tripeptide QGP (Figure 3, Table S1). These results suggest that the evolution of skipper FibH is not merely the result of a simple gene duplication event.

## 3. Discussion

In this study, we obtained and characterized 114 *FibH* sequences, providing the most systematic description of insect *FibH* to date. There are four significant findings. Firstly, we have completed the *FibH* sequences of many insects, such as bagworms (*Luffia ferchaultella*). Second, it was the first time that copy number variation events of the *FibH* gene were found in insects, occurring only in skippers. Third, we revealed that the FibH of skippers evolved from that of Papilionoidea. Lastly, we identified GAGAGS, polyAla, or polySer as the predominant motifs in Lepidopteran FibH, whereas amino acids Tyr, Val, Arg, and Trp are unique to the majority of Trichoptera FibH.

Lepidoptera comprises approximately 130 families, with a genome of about 2279 insect species from about 46 families being sequenced. However, many insect genomes are still incomplete with only contigs; thus, we were only able to extract the *FibH* gene from 40 families. In contrast, there is even less whole-genome data available for the Trichoptera. Scaling up the sequencing of Lepidoptera and Trichoptera, accelerating insect whole-genome assembly, and improving genome quality will further expedite research on the *FibH* gene, providing a more comprehensive and in-depth understanding of insect silk proteins.

The complexity and functionality of spider silk originate from the diversity of spidroins, including MaSp, MiSp, and PySp, among others. These spidroins have various variants, each with a unique structure, which imparts different physical and chemical properties to spider silk. Additionally, multiple spidroin variants can enhance silk protein synthesis efficiency, which is particularly important in extreme environments or rapid spider web construction [25,26,27,28,29]. Previously, there have been no reports on copy number variations of the insect *FibH* gene. In this study, we first discovered that some butterfly species have 2–3 copies of the *FibH* gene, with each copy having different isoelectric points and hydrophobicity, suggesting unique mechanical properties of these butterfly silks (Table 1). Silkworm silk exhibits different mechanical properties at each instar, primarily due to the varying proportions of three silk fibroins (FibH, FibL, and P25) in different instar silks. In butterflies, the relationship between an increased *FibH* copy number and the silk protein yield, synthesis rate, and mechanical properties of silk fibers still needs experimental verification.

BmFibH is synthesized by the silkworm posterior silk gland (PSG). The FibH forms a silk protein complex with FibL and P25 in a 6:6:1 ratio, and its secondary structure is mainly a random coil or *α*-helix in the PSG cell. When the silk protein is secreted into the silk gland lumen, during the transportation process from the PSG to the anterior silk gland (ASG), FibH undergoes changes in physical and chemical environments, causing its secondary structure to transition from a random coil or *α*-helix to an antiparallel *β*-sheet [17,18,19,20]. Near the spinneret of ASG, a highly ordered anisotropic herringbone pattern gradually forms, preparing for spinning silk [30]. To understand the structural differences of FibH across insects, we used Alphafold 2 to predict the three-dimensional structure of FibH and MaSp 1. BmorFibH, LhesMaSp 1, and MiSp (A6YP79.1.A) exhibit similar structures characterized by numerous relatively uniform helices, which may be related to strong selection pressures acting on silkworms and spiders. AalFibH (moth) and OalbFibH (caddisfly) share a similar structure with fewer non-uniform helices, and their NTD contains both *β*-sheets and *α*-helices. However, the NTD of PbarFibH (butterfly) also contains *β*-sheets and *α*-helices, but no uniform helical structure (Figure S4). It is worth mentioning that these structures are only predictions and need to be further experimentally verified.

Insect FibH is composed of numerous repetitive motifs (Figure 3, Table S1). The type, number, and arrangement of motifs determine the mechanical properties of the silk fibers. Spider dragline silk is stronger than silkworm silk, mainly due to the high uniformity of repetitive sequences and polyAla in MaSp. The FibH of bagworms contains both polyAla and GAGAGS motifs, making the mechanical properties of bagworm silk comparable to that of spider silk [31]. The most abundant motifs in the BmorFibH are hexapeptide GAGAGS and GAGAGY [13]. Yoko Takasu et al. [24] assembled these two motifs into a highly ordered artificial FibH and completely replaced the original *FibH* gene. The resulting homozygous individuals were unable to spin silk, suggesting that the design of silk proteins must follow natural principles. Artificial intelligence, with its powerful learning and computational capabilities, has been used for protein structure prediction and the development of new Cas proteins, among other applications [32,33,34,35]. It is also an inevitable trend to predict the structure of silk proteins, design silk proteins, and customize artificial silk [36,37,38]. A large database of silk protein sequences and mechanical properties’ data of silk fibers is fundamental for artificial intelligence to design silk proteins. Currently, there are about 500 silk protein sequences available, and many insect genomes are accessible [13,16,27]. However, what is most lacking at present are data on the mechanical properties of silk fibers. Collecting numerous silk fibers and measuring their mechanical properties to clarify the relationship between mechanical performance and the FibH sequence will help deep learning to accelerate the development and utilization of novel silk fibers.

All in all, our study develops a profound understanding of insect FibH, providing a broader perspective on the possible utility of these proteins in bioengineering and other apposite applications.

## 4. Materials and Methods

### 4.1. Extract the Complete FibH Gene Sequence from Database

Search in NCBI (https://www.ncbi.nlm.nih.gov/datasets/genome/) accessed on 27 March 2024 with the insect species name as a keyword. Genomes sequenced by PACBIO_SMRT (Sequel II or Revio) and with coverage depth above 20× are downloaded, while genomes sequenced by NGS and ONT are not utilized. Meanwhile, the 40 amino acids at the N- and C-terminus of the known FibH of *Bombyx mori*, *Ephestia kuehniella*, *Antheraea pernyi*, *Samia ricini*, *Antheraea yamamai*, *Antheraea assama*, and *Yponomeuta cagnagella* were used as tag sequences to blast the searched genome. Use the retrieved nucleotide sequences with homology to search the genome, determine the 5′ and 3′ terminus of the *FibH* gene, and extract the complete *FibH* gene sequence. Then, refer to the *FibH* genes of *Bombyx mori*, *Ephestia kuehniella*, *Antheraea pernyi*, *Samia ricini*, *Antheraea yamamai*, *Antheraea assama*, and *Yponomeuta cagnagella*, and finally determine the exons and introns of the *FibH* gene of the selected insect. The complete FibH protein sequence was deduced using Bioedit 7.2.

### 4.2. Data Analysis and Graphing

The theoretical isoelectric point (pI) and grand average of hydropathicity (GRAVY) of FibH were calculated by protparam (https://web.expasy.org/protparam/ accessed on 27 March 2024), and the signal peptide was predicted by SignalP-5.0 [39]. Multiple sequence alignment was performed using the online software mafft version 7 (https://mafft.cbrc.jp/alignment/server/ accessed on 27 March 2024). The multiple sequence alignment results and *FibH* gene mapping on the genome were visualized by TBtools-II v2.086 [13]. The phylogenetic tree was generated by IQ-TREE 2 [40] with maximum likelihood (ML) and bootstrap value 1000. Use Adobe Illustrator 2023 (Adobe Inc., Mountain View, CA, USA) and the online software ITol v6 (https://itol.embl.de/itol.cgi accessed on 27 March 2024) to beautify the phylogenetic tree. OriginPro 2021 (OriginLab, Northampton, MA, USA) is used to draw curve graphs and scatter plots. We first retrieve the FibH sequences using a sliding window with a width of 6 and count the most frequently occurring motif. Then, using the left boundary of this motif sequence, we divide the entire FibH sequence into sequence subsets. Each subset is then analyzed for motifs using MEME software (v5.5.5) with parameters (-minw 6 -maxw 100 -allw -mod anr -nmotifs 20). The motifs with occurrence sites ≥3 in the results are considered the motif set for that FibH sequence.

### 4.3. Three-Dimensional Structure Prediction of FibH

The 3D structure of FibH was predicted using online Alphafold 2 [41] (https://colab.research.google.com/github/sokrypton/ColabFold/blob/main/AlphaFold2.ipynb?authuser=3#scrollTo=KK7X9T44pWb7 accessed on 27 March 2024). The N-terminal 705 amino acids of FibH were used as a query sequence. Except for setting num_recycles to 12, other parameters are defaults.

## Figures and Tables

**Table 1:** The skipper *FibH* genes with multiple copies and their encoded protein.

Gene	Exon 1 (bp)	Intron (bp)	CDS (bp)	Protein (aa)	Signal Peptide (aa)	PI	Grand Average of Hydropathicity (GRAVY)	Top 5 Amino Acids
*BmorFibH*	42	971	15,792	5263	18	4.39	0.216	Gly, Ala, Ser, Tyr, Val
*HcomFibH 1*	42	1189	12,855	4284	18	9.19	−0.288	Ala, Gly, Pro, Gln, Ser
*HcomFibH 2*	42	4906	25,620	8539	18	8.85	−1.029	Gly, Pro, Gln, Ala, Ser
*TactFibH 1*	42	1338	20,112	6703	18	4.89	−0.323	Gly, Ala, Pro, Ser, Gln
*TactFibH 2*	42	1493	26,265	8754	18	7.87	−0.153	Gly, Ala, Pro, Ser, Gln
*TactFibH 3*	42	2878	24,417	8138	18	7.85	−1.256	Gly, Pro, Gln, Ala, Ser
*TsylFibH 1*	42	1648	13,863	4620	18	5.29	−0.104	Gly, Ala, Ser, Pro, Gln
*TsylFibH 2*	42	2491	20,706	6901	18	9.14	−0.155	Ala, Gly, Pro, Ser, Gln
*TsylFibH 3*	42	2718	20,454	6817	18	8.14	−1.178	Gly, Gln, Pro, Ala, Ser
*CpalFibH 1*	42	3349	16,341	5446	18	6.74	−0.977	Gly, Pro, Gln, Ala, Ser
*CpalFibH 2*	42	933	13,422	4473	18	5.02	−0.395	Ala, Gly, Ser, Pro, Tyr

## Data Availability

Data is contained within the article and Supplementary Material.

## Supplementary Materials

The following supporting information can be downloaded at: https://www.mdpi.com/article/10.3390/ijms25137179/s1.
