# Supplementary material for: Overview and Evolution of Insect Fibroin Heavy Chain (FibH)
Source: Int J Mol Sci. 2024 Jun 29;25(13):7179. doi: 10.3390/ijms25137179 (PMC11241164; doi:10.3390/ijms25137179)
Supplement: Supplementary file 1 [file ijms-25-07179-s001.zip › Supplementary materials.pdf]

Supplementary materials

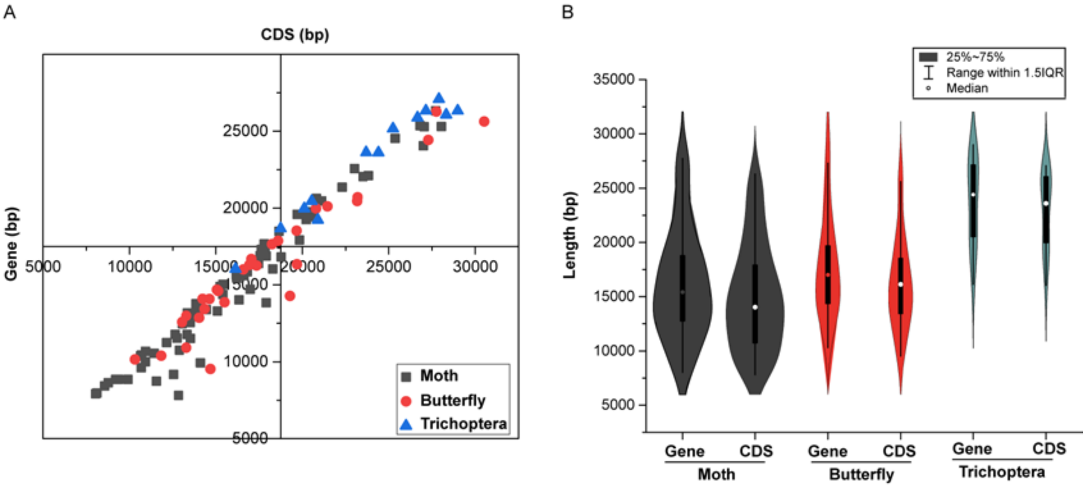

Fig. S1 Length of insect *FibH* gene and their CDS. (A) Correlation analysis of *FibH* genes and their CDSs in moth, butterfly, and Trichoptera. (B) Length statistics of *FibH* genes and their CDSs in moth, butterfly, and Trichoptera.

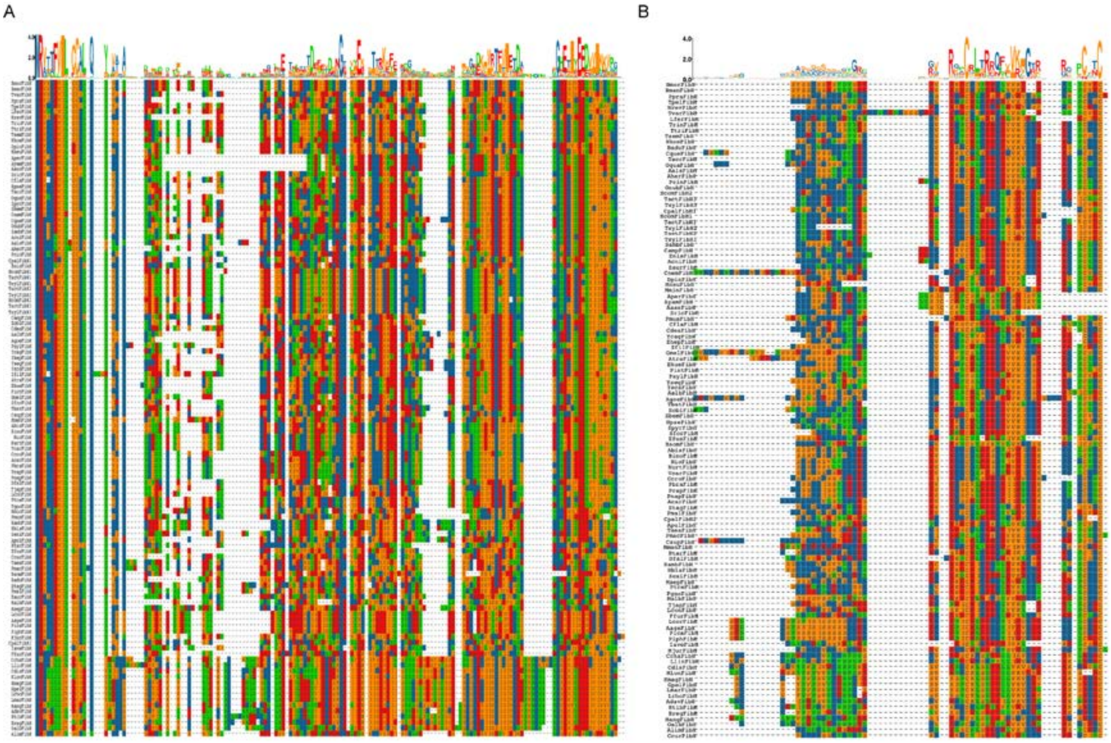

Fig. S2 Sequence alignment of N- and C- terminus of insect FibH. (A) N-terminus sequence alignment of FibH. (B) C-terminus sequence alignment of FibH.

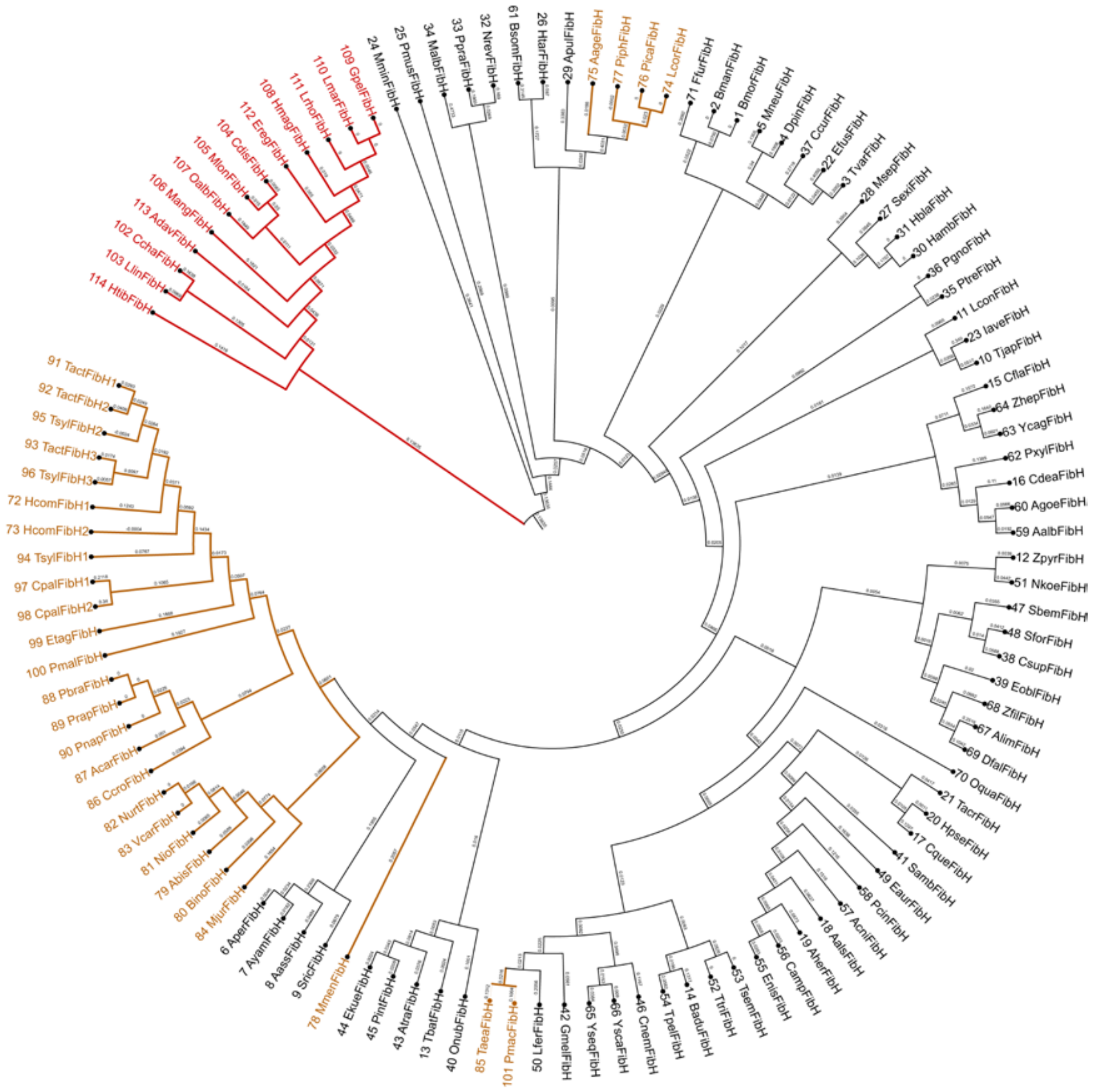

Fig. S3 Phylogenetic tree of the insect FibH.

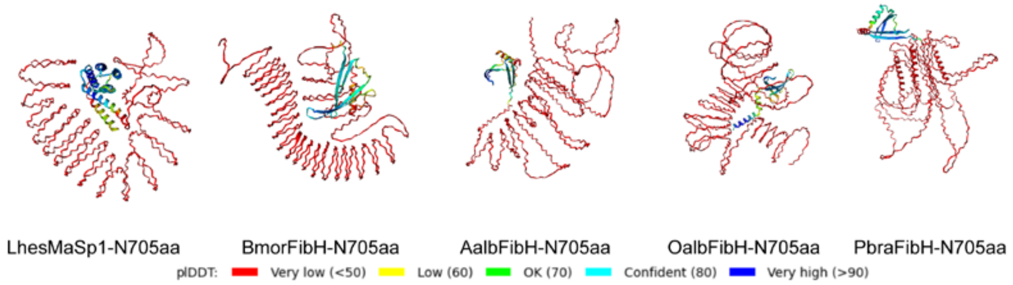

Fig. S4 The 3D structures of the N-terminus and part of the repetitive sequence (705 amino acids) of FibH of *Bombyx mori*, *Argyrethia albistria*, *Luffia ferchaultella*, *Odontocerum albicorne*, *Pieris brassicae*, and *Latrodectus Hesperus MaSp1* (LhesMaSp1) predicted by Alphafold 2.
